# Reverse Regioselective Dicarbofunctionalization via *Anti*‐Michael‐Type Addition

**DOI:** 10.1002/anie.202505391

**Published:** 2025-04-21

**Authors:** Hirotsugu Suzuki, Ryota Moro, Takanori Matsuda

**Affiliations:** ^1^ Tenure‐Track Program for Innovative Research University of Fukui 3‐9‐1 Bunkyo Fukui‐shi Fukui 910‐8507 Japan; ^2^ Department of Applied Chemistry Tokyo University of Science 1‐3 Kagurazaka Shinjuku‐ku Tokyo 162‐8601 Japan

**Keywords:** *Anti*‐Michael‐type addition, Dicarbofunctionalization, Directing group, Palladium, Reverse regioselectivity

## Abstract

Vicinal dicarbofunctionalization of α,β‐unsaturated carbonyl compounds is a classical yet versatile method for constructing complex molecular architectures in a single step. However, the regioselectivity is typically governed by the electronic properties of the alkene moiety, leading to the introduction of a nucleophile at the β‐position and an electrophile at the α‐position. Herein, we report a palladium‐catalyzed dicarbofunctionalization of acrylamides via *anti*‐Michael‐type addition, achieving reverse regioselectivity relative to traditional approaches. This strategy enables the efficient incorporation of various (hetero)arene nucleophiles at the α‐position and carbon electrophiles, including iodoarenes, vinyl iodides, and iodomethane, at the β‐position to furnish dicarbofunctionalized amides in good yields. Mechanistic investigations suggest that the reaction proceeds through an alkylpalladium intermediate formed via the α‐addition of a nucleophile.

## Graphical abstract

A reverse regioselective dicarbofunctionalization of acrylamides via *anti*‐Michael‐type addition is reported. Various (hetero)arene nucleophiles and carbon electrophiles were incorporated in a manner opposite to conventional regioselectivity. A key feature of this reaction is the formation of an alkylpalladium intermediate, which enables the achievement of the uncommon regioselectivity.

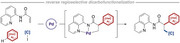

Vicinal dicarbofunctionalization (DCF) is a powerful strategy in organic synthesis, enabling the simultaneous formation of two carbon–carbon (C─C) bonds in a single step.^[^ ^1^, ^2^, ^3^, ^4^, ^5^, ^6^, ^7^ ^]^ Among various substrates, the DCFs of α,β‐unsaturated carbonyl compounds have garnered significant attention due to the inherent versatility of the carbonyl group,^[^ ^8^, ^9^, ^10^, ^11^ ^]^ which is widely prevalent and highly amenable to further transformations.^[^ ^12^, ^13^, ^14^ ^]^ Traditionally, DCF is achieved via the Michael addition of an organometallic reagent to an α,β‐unsaturated carbonyl compound, followed by trapping the resulting enolate intermediate with an electrophile (Scheme 1a).^[^ ^8^, ^9^, ^10^, ^11^ ^]^ This approach consistently introduces the nucleophile at the β‐position and the electrophile at the α‐position. However, achieving regioselectivity opposite to this conventional pattern remains a significant challenge within the traditional mechanistic framework.^[^ ^15^, ^16^ ^]^ Overcoming this limitation requires a novel synthetic strategy capable of circumventing the inherent regioselectivity of conventional DCF, which is governed by the electronic properties.

**Scheme 1:**
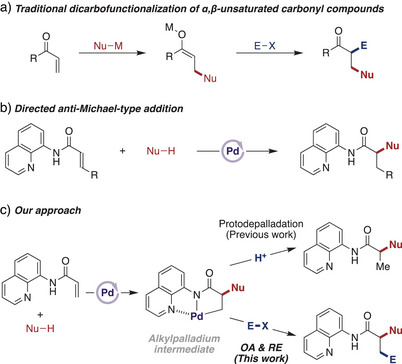
Traditional DCF and our approach to reverse regioselective DCF.

*anti*‐Michael‐type addition is a distinctive bond‐forming reaction that facilitates the introduction of a nucleophile at the α‐position of α,β‐unsaturated carbonyl compounds.^[^ ^17^, ^18^, ^19^, ^20^, ^21^, ^22^, ^23^, ^24^, ^25^, ^26^ ^]^ Despite its significant synthetic potential, achieving this unusual regioselectivity has proven challenging due to the inherent electronic bias of the activated alkene moiety. To address this challenge, we recently developed a palladium‐catalyzed *anti*‐Michael‐type addition of (hetero)arenes and 2‐pyridones, enabled by an 8‐aminoquinolyl directing group (Scheme 1b).^[^ ^17^, ^18^ ^]^ Preliminary mechanistic studies suggested that this reaction proceeds via the formation of an alkylpalladium intermediate, following nucleophilic addition at the α‐position (Scheme 1c).

Inspired by recent advancements in palladium‐catalyzed directed difunctionalization of unactivated alkenes, as reported by Engle et al.^[^ ^27^, ^28^, ^29^, ^30^, ^31^, ^32^ ^]^ and others,^[^ ^33^, ^34^, ^35^ ^]^ we hypothesized that intercepting this alkylpalladium intermediate with a carbon electrophile,^[^ ^36^, ^37^, ^38^, ^39^, ^40^, ^41^, ^42^, ^43^, ^44^ ^]^ rather than a proton, could provide a novel synthetic strategy for reverse regioselective DCF of acrylamides. While a similar product can be obtained using our previously reported *anti*‐Michael‐type addition with a β‐substituent on the acrylamide substrate,^[^ ^17^ ^]^ this DCF approach offers several synthetic advantages. First, it enhances step economy and overall yield by shortening the reaction sequence starting from commercially available materials. Second, its modularity allows for the synthesis of a diverse array of chemical structures in fewer steps. Third, several β‐substituents that were not tolerated in previous *anti*‐Michael‐type additions, such as heteroaryl and vinyl groups, can be introduced through the DCF process. Herein, we report a reverse regioselective DCF of *N*‐(quinolin‐8‐yl)acrylamide, employing a strategy involving *anti*‐Michael‐type addition followed by interception of the alkylpalladium intermediate (Scheme 1c). This transformation demonstrates broad functional group compatibility, efficiently incorporating various electron‐rich arenes, as well as aryl, vinyl, and methyl iodides.

Based on our previous work on the *anti‐*Michael‐type addition of (hetero)arenes,^[^ ^17^, ^18^ ^]^ we initiated our investigation using *N*‐(quinolin‐8‐yl)acrylamide (**1a**), 1‐methylindole (**2a**), and 4‐iodoanisole (**3a**) as model substrates (Table 1). Our initial attempt employed Pd(OCOCF_3_)_2_ (10 mol%) as the catalyst, with K_2_CO_3_ (1.0 equiv.) as the base in CH_3_CN (1.0 M) at 120 °C for 18 h. Gratifyingly, a trace amount of the desired product **4a** was observed (Entry 1), prompting extensive optimization of the reaction conditions. The choice of a base was critical in enhancing the chemical yield of **4a** (Entries 1–3), with KHCO_3_ providing the best results (Entry 2). Notably, Pd(OAc)_2_ afforded a slight improvement in yield compared to Pd(OCOCF_3_)_2_, contrary to our earlier observations (Entry 4).^[^ ^17^, ^18^ ^]^ In contrast, other palladium complexes, including Pd(0) species, led to inferior yields (Entry 5). Lowering the reaction temperature to 100 °C provided a modest improvement (Entry 6). Reaction concentration also had a substantial effect, with an 83% isolated yield of **4a** achieved in a 2.0 M CH_3_CN solution (Entry 7). This reaction showed slight sensitivity to moisture and atmospheric oxygen, as evidenced by reduced yields in the presence of H_2_O or under an air atmosphere (Entries 8 and 9). Control experiments confirmed the necessity of both the palladium catalyst and base for the reaction to proceed (Entries 10 and 11).

**Table 1:** Optimization of reaction conditions^a)^

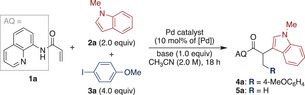					
Entry	Pd catalyst	Base	Temp (°C)	Yield (%, **4a**)^b)^	Yield (%, **5a**)^b)^
1^c)^	Pd(OCOCF_3_)_2_	K_2_CO_3_	120	3	0
2^c)^	Pd(OCOCF_3_)_2_	KHCO_3_	120	59	2
3^c)^	Pd(OCOCF_3_)_2_	K_3_PO_4_	120	31	trace
4^c)^	Pd(OAc)_2_	KHCO_3_	120	61	17
5^c)^	Pd_2_(dba)_3_	KHCO_3_	120	15	trace
6^c)^	Pd(OAc)_2_	KHCO_3_	100	66	16
7	Pd(OAc)_2_	KHCO_3_	100	79 (83)	12
8^d)^	Pd(OAc)_2_	KHCO_3_	100	66	3
9^e)^	Pd(OAc)_2_	KHCO_3_	100	72	11
10	–	KHCO_3_	100	0	0
11	Pd(OAc)_2_	–	100	14	8

^a)^ Reaction conditions: **1a** (0.4 mmol), **2a** (0.8 mmol), **3a** (1.6 mmol), Pd catalyst (10 mol% of [Pd]), and base (0.4 mmol) were reacted in CH_3_CN (0.2 mL) for 18 h, unless otherwise noted.

^b)^ Yield was determined by ^1^H NMR analysis using 1,2,4,5‐tetramethylbenzene as an internal standard. The value in parentheses indicates isolated yield.

^c)^ The reaction was conducted on a 0.2 mmol scale. The concentration of the solution is 1.0 M.

^d)^ H_2_O (2.0 equiv.) was added.

^e)^ The reaction was conducted under an air atmosphere.

Next, we examined the scope of the DCF under the optimized reaction conditions (Scheme 2). Using *N*‐benzylindole as a substrate afforded **4b** in 89% yield. However, alternative *N*‐substituents, such as allyl and phenyl, significantly reduced the yields, giving **4c** and **4d** in 40% and 32% yields, respectively. The reaction with *N*‐unprotected indole was less effective, producing **4e** in only 13% yield. The influence of substituents on the benzenoid moiety was also explored. Electron‐donating and ‐withdrawing groups exhibited minimal impact on the reaction efficiency, regardless of their positions, delivering **4f**–**o** in 51%–77% yields. A notable exception was observed with a 4‐methylindole substrate, which failed to yield the desired product, likely due to steric hindrance near the nucleophilic site. Expanding the substrate scope further, other electron‐rich heteroarenes, such as pyrroles and a thiophene, underwent the reaction to produce **4p**–**r** in 29%–47% yields. Similarly, electron‐rich arenes proved compatible, affording **4s** and **4t** in 31% and 50% yields, respectively.

**Scheme 2:**
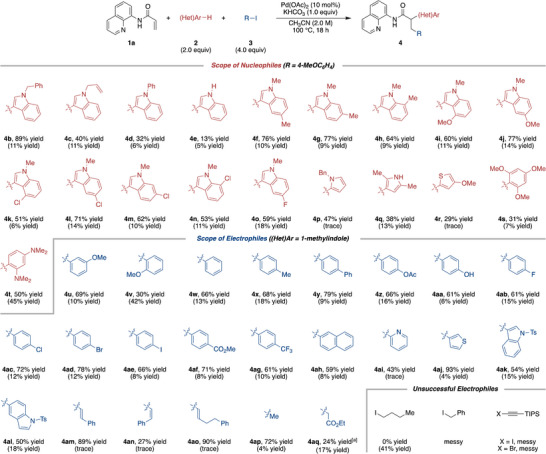
Substrate scope of reverse regioselective DCF. Reaction conditions: **1a** (0.4 mmol), **2** (0.8 mmol), **3** (1.6 mmol), Pd(OAc)_2_ (10 mol%), and KHCO_3_ (0.4 mmol) were reacted in CH_3_CN (0.2 mL) at 100 °C for 18 h. The values in parentheses indicate the NMR yields of *anti*‐Michael adducts **5**. a) Ethyl bromoacetate was used as an electrophile.

The scope of carbon electrophiles was then investigated. While 3‐iodoanisole proved to be an effective coupling partner, yielding **4u** in 69% yield, 2‐iodoanisole showed a marked decrease in efficiency (**4v**). In the latter case, a significant amount of the *anti*‐Michael adduct **5** was observed, likely due to the slower oxidative addition step. Nonetheless, a range of electron‐rich and ‐deficient iodoarenes successfully delivered products **4w**–**ag** with 61%–79% yields. Notably, the synthesis of **4aa** demonstrated the superiority of this DCF approach, reducing the reaction sequence from five to two steps and increasing the overall yield from 21% to 52% compared to the previously reported *anti*‐Michael‐type addition.^[^ ^17^, ^18^, ^45^ ^]^ Polyaromatic and heteroaromatic electrophiles were also compatible, producing **4ah**–**al** in 43%–93% yields. Even substrates with strongly coordinating groups, which can inhibit the reaction under the optimized conditions^[^ ^27^ ^]^ as well as in *anti*‐Michael‐type addition,^[^ ^45^ ^]^ were tolerated (**4ai**). Vinyl iodides served as effective electrophiles as well, yielding **4am** and **4ao** in 89% and 90% yields, respectively. However, using (*Z*)‐alkene resulted in a significant drop in yield to 27% (**4an**). Although the yield of **4an** was moderate, these results further emphasize the advantage of this DCF approach, as such structures were not accessible via the corresponding *anti*‐Michael‐type addition.^[^ ^45^ ^]^ Remarkably, this methodology also enabled the introduction of a methyl group at the β‐position (**4ap**). Additionally, ethyl bromoacetate was accommodated under the reaction conditions, delivering **4aq** in 24% yield. However, other alkyl iodides, such as 1‐iodobutane and benzyl iodide, failed to provide the corresponding products. Similarly, attempts to introduce an alkyne moiety at the β‐position were unsuccessful. While this reverse regioselective DCF method has certain substrate limitations, it provides a versatile approach for synthesizing a wide array of 2,3‐disubstituted propionamides with improved efficiency and yields.

To demonstrate the utility of this methodology, a gram‐scale reaction and subsequent derivatization of **4a** were carried out (Scheme 3).^[^ ^46^ ^]^ Scaling up the reaction with 4 mmol of **1a** produced **4a** in 73% yield (1.27 g). Hydrolysis of the quinolylamide moiety afforded carboxylic acid **6** in an excellent 96% yield.

**Scheme 3:**
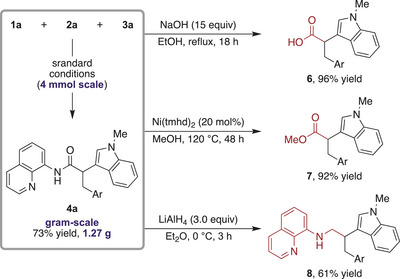
Gram‐scale reaction and transformation of **4a**. Ar = 4‐MeOC_6_H_4_. Ni(tmhd)_2_; Nickel(II) bis(2,2,6,6‐tetramethyl‐3,5‐heptanedionate).

Similarly, treatment of **4a** with a catalytic amount of Ni(tmhd)_2_ resulted in the formation of methyl ester **7** in 92% yield.^[^ ^47^ ^]^ Finally, reduction of **4a** afforded the corresponding amine **8** in 61% yield.

To elucidate the reaction mechanism, several control experiments were performed. Initially, we envisioned three possible mechanistic scenarios for the DCF, as depicted in Scheme 4a. The first scenario involves an initial *anti*‐Michael‐type addition, generating the *anti*‐Michael adduct **5**, followed by C(sp^3^)–H bond activation to furnish the difunctionalized product **4** (Pathway 1).^[^ ^36^, ^37^, ^38^, ^39^, ^40^, ^41^, ^42^, ^43^, ^44^ ^]^ In the second scenario, β‐substituted acrylamides **9** or **10** are formed via a Heck reaction^[^ ^48^, ^49^, ^50^, ^51^ ^]^ or C(sp^2^)─H bond activation of acrylamide **1a**,^[^ ^52^, ^53^ ^]^ followed by an *anti*‐Michael‐type addition to yield the product **4** (Pathway 2). The third scenario proposes that *anti*‐Michael‐type addition produces an alkylpalladium intermediate **A**, which undergoes oxidative addition with the carbon electrophile to form palladium(IV) intermediate **B**. Subsequent reductive elimination from intermediate **B** delivers the difunctionalized product **4** (Pathway 3).^[^ ^27^, ^28^, ^29^, ^30^, ^31^, ^32^, ^33^, ^34^, ^35^ ^]^

**Scheme 4:**
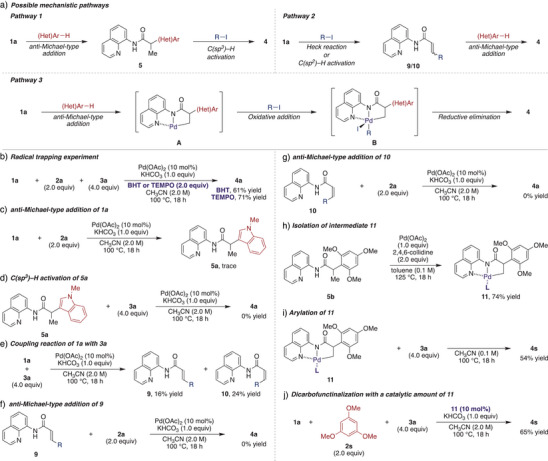
Mechanistic investigation. R = 4‐MeOC_6_H_4_, **L** = 2,4,6‐collidine.

Given that recent studies on reverse regioselective DCF suggest that these reactions proceed via a single‐electron pathway,^[^ ^15^, ^16^ ^]^ radical trapping experiments were conducted using radical inhibitors, BHT or TEMPO, under the standard reaction conditions. However, no inhibition of the reaction was observed in either case, indicating that this reverse regioselective DCF follows a two‐electron pathway (Scheme 4b). Further experiments were conducted to evaluate the plausibility of Pathway 1. *anti*‐Michael‐type heteroarylation of acrylamide **1a** was attempted under the standard conditions, but only a trace amount of the product was observed (Scheme 4c). This finding aligns with our earlier observation that the addition of an alkaline metal base halts the *anti*‐Michael‐type addition process.^[^ ^17^ ^]^ Moreover, an investigation into the cleavage of the C(sp^3^)─H bond in the *anti*‐Michael adduct **5a** did not yield the difunctionalized product **4a** (Scheme 4d), rendering Pathway 1 unlikely. Pathway 2 was then examined. A coupling reaction between acrylamide **1a** and 4‐iodoanisole **3a** resulted in the formation of β‐substituted acrylamides **9** and **10** (Scheme 4e).^[^ ^52^, ^54^ ^]^ However, subsequent *anti*‐Michael‐type additions of amides **9** or **10** did not proceed under standard conditions (Scheme 4f,g). These results suggest that Pathway 2 is also improbable. Finally, experiments were conducted to investigate the plausibility of Pathway 3. Treatment of *anti*‐Michael adduct **5b** with Pd(OAc)_2_ and 2,4,6‐collidine produced alkylpalladium complex **11** in 74% yield (Scheme 4h).^[^ ^55^ ^]^ A stoichiometric reaction of complex **11** with 4‐iodoanisole **3a** afforded the difunctionalized product **4s** in 54% yield. Furthermore, replacing Pd(OAc)_2_ with a catalytic amount of complex **11** under standard conditions delivered **4s** in 65% yield. These results strongly support that the reverse regioselective DCF proceeds via the formation of intermediate **A**, with Pathway 3 being the most plausible mechanism at this stage.

In conclusion, we have developed a palladium‐catalyzed dicarbofunctionalization of *N*‐(quinolin‐8‐yl)acrylamide via *anti*‐Michael‐type addition. This reaction offers a practical and efficient strategy for achieving reverse regioselectivity, enabling the introduction of a nucleophile at the α‐position and an electrophile at the β‐position. Compared to previous *anti*‐Michael‐type addition methods, this protocol enhances synthetic efficiency by reducing the total number of reaction steps and improving overall yield. Mechanistic studies revealed that the reaction proceeds via the formation of an alkylpalladium intermediate through α‐addition of the nucleophile, followed by sequential oxidative addition and reductive elimination. Given the limited examples of reverse regioselective difunctionalization of α,β‐unsaturated carbonyl compounds, this methodology not only addresses a significant synthetic challenge but also provides a versatile approach for constructing structurally diverse molecules that are difficult to access with traditional dicarbofunctionalization techniques.

## Supporting Information

The authors have cited additional references within the Supporting Information.^[^ ^56^, ^57^, ^58^, ^59^ ^]^

## Conflict of Interests

The authors declare no conflict of interest.

## Supporting information



Supporting Information

## Data Availability

The data that support the findings of this study are available in the supplementary material of this article.
