# Supplementary material for: Reverse Regioselective Dicarbofunctionalization via Anti‐Michael‐Type Addition
Source: Angew Chem Int Ed Engl. 2025 Apr 21;64(25):e202505391. doi: 10.1002/anie.202505391 (PMC12171322; doi:10.1002/anie.202505391)
Supplement: Supplementary file 1 — Supporting Information [file ANIE-64-e202505391-s001.docx]

**Supporting Information**

**Bimetallic Designing Based on the Nucleation Rate of Prussian Blue Analogues for Enhanced Aqueous Zinc Ions Batteries with Glycerol-Water Hybrid Electrolytes**

Biao Yang,^[a,b,+]^ Yuxin Shi,^[b,+]^, Gongjing Song,^[b]^ Chengang Pei,^[b]^ Yan Yan,^[b]^ Zhidong Chen,^[a*]^ and Huan Pang^[b*]^

*[a].* *School of Materials Science & Engineering**, Changzhou University, Changzhou, 213164 Jiangsu, (P. R. China)*

*E-mail:* *zdchen@cczu.edu.cn*

*[b]. School of Chemistry and Chemical Engineering*

*Yangzhou University, Yangzhou, 225002 Jiangsu, (P. R. China)*

*E-mail:* *[huanpangchem@hotmail.com](mailto:huanpangchem@hotmail.com)*

**Keywords**: Structural regulation, Glycerol-water hybrid electrolytes, Heterostructures, Prussian blue analogues, Aqueous zinc-ion batteries,

**Experiment section**

**1. Chemicals**

All of the reagents used in this experiment were analytical grade and they used without further purifications. All chemicals, Cobaltous chloride (CoCl_2_·6H_2_O), Copper(II) chloride (CuCl_2_·2H_2_O), Manganese(II) chloride (MnCl_2_·2H_2_O), Nickel chloride (NiCl_2_·2H_2_O), Ferric trichloride (FeCl_3_·6H_2_O), Zinc chloride (ZnCl_2_·2H_2_O), Cupric acetate (Cu(COOCH_3_)_2_·H_2_O), Cobalt(II) acetate (Co(COOCH_3_)_2_·4H_2_O), Manganous acetate (Mn(COOCH_3_)_2_·4H_2_O), Nickelous acetate (Ni(COOCH_3_)_2_·4H_2_O), potassium hexacyanoferrate(III) (K_3_[Fe(CN)_6_]) and hydrochloric acid (HCl, 36.0-38.0%) were purchased from Shanghai Sinopharm Chemical Reagent Co. Ltd., China. All the above were used as analytical-grade reagents without further purification. All aqueous solutions were prepared with high-purity deionized water (18 MΩ cm^-1^).

**2. Materials synthesis**

**2.1 Synthesis of V_6_O_13_**

In a typical process, 0.4 g of V_2_O_5_ was added to a mixed solution of C_2_H_5_OH and deionized water (volume ratio is 5:4) under strong stirring until the solution was dispersive evenly. The mixed solution was transferred to a 100 mL autoclave, sealed, and kept at 160 °C for 24 h. After cooling to room temperature, the pristine products were obtained through centrifugation with deionized water and ethanol.

**2.2 Synthesis of diluted hydrochloric acid**

The 0.6 mL hydrochloric acid (36.0-38.0%) was diluted to 4 mL.

**2.3 Synthesis of VCo-PBA**

0.025 g V_6_O_13_ were dissolved in 4 mL diluted hydrochloric acid, and then 0.0039 g CoCl_2_·6H_2_O was added to generate clear solution A. Then, 0.1580 g K_3_[Fe(CN)_6_ was dissolved in 10 mL of deionized water to form solution B. Then, solution B was added into solution A under stirring at room temperature for 24 h at room temperature. The precipitate was washed via several rinsing-centrifugation cycles with deionized water and ethanol and then dried at room temperature. The obtained product was designated VCo-PBA.

**2.4 Synthesis of VCu-PBA**

0.025 g V_6_O_13_ was dissolved in 4 mL diluted hydrochloric acid, and then 0.0052 g CuCl_2_·6H_2_O was added to generate clear solution A. Then, 0.1580 g K_3_[Fe(CN)_6_ was dissolved in 10 mL of deionized water to form solution B. Then, solution B was added into solution A under stirring at room temperature for 24 h at room temperature. The precipitate was washed via several rinsing-centrifugation cycles with deionized water and ethanol and then dried at room temperature. The obtained product was designated VCu-PBA.

**2.5 Synthesis of VMn-PBA**

0.025 g V_6_O_13_ was dissolved in 4 mL diluted hydrochloric acid, and then 0.0060 g MnCl_2_·6H_2_O was added to generate clear solution A. Then, 0.1580 g K_3_[Fe(CN)_6_ was dissolved in 10 mL of deionized water to form solution B. Then, solution B was added into solution A under stirring at room temperature for 24 h at room temperature. The precipitate was washed via several rinsing-centrifugation cycles with deionized water and ethanol and then dried at room temperature. The obtained product was designated VMn-PBAs.

**2.6 Synthesis of VNi-PBA**

0.025 g V_6_O_13_ was dissolved in 4 mL diluted hydrochloric acid, and then 0.0073 g NiCl_2_·6H_2_O was added to generate clear solution A. Then, 0.1580 g K_3_[Fe(CN)_6_ was dissolved in 10 mL of deionized water to form solution B. Then, solution B was added into solution A under stirring at room temperature for 24 h at room temperature. The precipitate was washed via several rinsing-centrifugation cycles with deionized water and ethanol and then dried at room temperature. The obtained product was designated VNi-PBA.

**2.7 Synthesis of VFe-PBA**

0.025 g V_6_O_13_ was dissolved in 4 mL diluted hydrochloric acid, and then 0.0081 g FeCl_3_·6H_2_O was added to generate clear solution A. Then, 0.1580 g K_3_[Fe(CN)_6_ was dissolved in 10 mL of deionized water to form solution B. Then, solution B was added into solution A under stirring at room temperature for 24 h at room temperature. The precipitate was washed via several rinsing-centrifugation cycles with deionized water and ethanol and then dried at room temperature. The obtained product was designated VFe-PBA.

**2.8 Synthesis of VZn-PBA**

0.025 g V_6_O_13_ was dissolved in 4 mL diluted hydrochloric acid, and then 0.0041 g MnCl_2_·6H_2_O was added to generate clear solution A. Then, 0.1580 g K_3_[Fe(CN)_6_ was dissolved in 10 mL of deionized water to form solution B. Then, solution B was added into solution A under stirring at room temperature for 24 h at room temperature. The precipitate was washed via several rinsing-centrifugation cycles with deionized water and ethanol and then dried at room temperature. The obtained product was designated VZn-PBA.

**2.9 Preparation of M-PBAs (M=Cu, Co, Mn, Ni)**

2 mmol acetate (Cu(COOCH_3_)_2_·H_2_O or Co(COOCH_3_)_2_·4H_2_O, Mn(COOCH_3_)_2_·4H_2_O or Ni(COOCH_3_)_2_·4H_2_O) and 2.25 mmol Na_3_C_6_H_5_O_7_·2H_2_O were dissolved in 50 mL deionized water to produce clear solution A. 2 mmol K_3_[Fe(CN)_6_] was dissolved in 50 mL deionized water to obtain clear solution B. Afterward, the solution B was directly poured into the solution A under stirring at room temperature. After continuous stirring for 10 minutes, the obtained mixed solution was aged at room temperature for 24 h. The products, including Co-PBA, Cu-PBA and Ni-PBA, were collected by centrifugation and washed repeatedly with deionized water and ethanol, respectively. Hence, Ni-PBA sample was prepared using 200 mL H_2_O in solution A and solution B, respectively.

**2.10 Preparation of glycerol-water hybrid electrolytes (GEs)**

Dissolving 3 mol Zn(OTf)_2_ in 1 kg of deionized water/glycerol hybrid solvent with an glycerol weight ratio of 50%, 40% and 20%, which are named as GE-5, GE-4 and GE-2, respectively.

**3. Materials characterization**

The morphology and size of the particles were characterized by field emission scanning electron microscope (SEM, Zeiss-Supra55), transmission electron microscope (TEM, JEM-1011), high resolution TEM (HRTEM, Tecnai G2 F30 S-TWIN), as well as energy dispersive X-ray spectrometry (EDS) mapping. The phase analysis of the as-prepared samples was tested by X-ray diffraction (XRD) in the 2θ range 5^o^-80^o^ range of 10^o^ min^-1^ on a Bruker D8 Advanced X-ray Diffractometer with Cu-Kα radiation (λ =0.15406 nm). Fourier transform infrared (FT-IR) transmission spectra were obtained on a BRUKER-EQUINOX-55 IR spectrophotometer. To understand the chemical state of the central atom, X-ray photoelectron spectroscopy (XPS) analysis with a standard monochromatic Al-Kα source was used. Additionally, thermogravimetry analysis (TGA) was performed by a PerkinElmer Pyris 1 TGA instrument under N_2_ atmosphere from 20 ^o^C to 400 ^o^C at a heating rate of 10 ^o^C min^-1^. At last, N_2_ adsorption measurements were performed on Quantachrome Instruments and the samples were pretreated at 80 ^o^C for 6h.

**4. Electrochemical Measurements**

The electrochemical performances were measured in 2032 coin-type cells. The working electrodes were prepared by mixing polyvinylidene fluoride (PVDF, 10 wt.%), Acetylene black (20 wt.%) and active materials (70 wt.%) in N-methyl-2 pyrrolidone (NMP) solvent on titanium foil which was used as the current collectors. The coated electrode was dried in vacuum at 60 ^o^C for 12 h. The separator was glass fiber. Statistically, the load quality of the active material is 0.5-0.8 mg, the thickness of the zinc foil is 0.1 mm, and the electrode is circular with a diameter of 12 mm. The cycling performance and rate capability were tested by a battery measurement system (CT2001A, Wuhan Land, China) at room temperature. The cyclic voltammetry (CV) and electrochemical impedance spectroscopy (EIS) were tested on a CHI 760E electrochemical workstation, and the CV curves was obtained in the potential range from 0.2 V to 2.0 V. The cycling performance of Zn/Zn symmetrical cell was tested at 1 mA cm^-2^ with the capacity of 1 mAh cm^-2^. The Tafel curves and Linear Sweep Voltammetry (LSV) curves were tested with the Zn anode and stainless steel as cathode.

**Figure S1.** Optical images of VM-PBA. (a) VCo-PBA. (b) VCu-PBA. (c) VMn-PBA. (d) VNi-PBA. (e) VFe-PBA. (f) VZn-PBA.

**Figure S2.** SEM images of VM-PBA. (a) VCo-PBA. (b) VCu-PBA. (c) VMn-PBA. (d) VNi-PBA. (e) VFe-PBA. (f) VZn-PBA.

**Figure S3.** TEM images of VM-PBA. (a) VCo-PBA. (b) VCu-PBA. (c) VMn-PBA. (d) VNi-PBA. (e) VFe-PBA. (f) VZn-PBA.

**Figure S4.** The particle size distribution of as prepared materials. (a) VCo-PBA. (b) VCu-PBA. (c) VMn-PBA. (d) VNi-PBA. (e) VFe-PBA. (f) VZn-PBA.

**Figure S5.** (a-f) The optical photos and corresponding SEM images of products at different stages of VCu-PBA. (g and h) The ultraviolet and visible (UV) spectrum of the supernatant solution at different reaction time. (i) The ICP-MS test of the supernatant solution at different reaction time.

**Figure S6.** The optical photos and corresponding SEM images of products at different stages of VCo-PBA. (g and h) The ultraviolet and visible (UV) spectrum of the supernatant solution at different reaction time. (i) The ICP-MS test of the supernatant solution at different reaction time.

**Figure S7.** The optical photos and corresponding SEM images of products at different stages of VNi-PBA. (g and h) The ultraviolet and visible (UV) spectrum of the supernatant solution at different reaction time. (i) The ICP-MS test of the supernatant solution at different reaction time.

**Figure S8.** The optical photos and corresponding SEM images of products at different stages of VZn-PBA. (g and h) The ultraviolet and visible (UV) spectrum of the supernatant solution at different reaction time. (i) The ICP-MS test of the supernatant solution at different reaction time.

**Figure S9.** The optical photos and corresponding SEM images of products at different stages of VMn-PBA. (g and h) The ultraviolet and visible (UV) spectrum of the supernatant solution at different reaction time. (i) The ICP-MS test of the supernatant solution at different reaction time.

**Figure S10.** The optical photos and corresponding SEM images of products at different stages of VFe-PBA. (g and h) The ultraviolet and visible (UV) spectrum of the supernatant solution at different reaction time. (i) The ICP-MS test of the supernatant solution at different reaction time.

**Figure S11**. ICP-MS data of the as prepared VM-PBAs materials. M represents another metal element of Co, Cu, Mn, Ni, Zn in VCo-PBA, VCu-PBA, VMn-PBA, VNi-PBA and VZn-PBA.

**Table S1.** The ICP data of mole percentage of the obtained VM-PBAs.

| Sample | V | Fe | Metal |
| --- | --- | --- | --- |
| VCo-PBA | 55.13% | 36.45% | 8.42% (Co) |
| VCu-PBA | 54.65% | 38.03% | 7.32% (Cu) |
| VMn-PBA | 50.6% | 38.29% | 11.11% (Mn) |
| VNi-PBA | 48.78% | 37.38% | 13.84% (Ni) |
| VFe-PBA | 40.78% | 59.22% |  |
| VZn-PBA | 13.27% | 41.53% | 45.2% (Zn) |

**Figure S12.** TGA curves of VM-PBAs. (a) VCo-PBA. (b) VCu-PBA. (c) VMn-PBA. (d) VNi-PBA. (e) VFe-PBA. (f) VZn-PBA.

**Figure S13.** N_2_ adsorption and desorption isotherms and corresponding pore-size distribution of VM-PBAs. (a) VCo-PBA. (b) VCu-PBA. (c) VMn-PBA. (d) VNi-PBA. (e) VFe-PBA. (f) VZn-PBA.

**Table S2.** The data of ICP test and corresponding formulas of the obtained VM-PBAs.

| Sample | Vanadium ion/  mol L^-1^ | Ferrum ions/  mol L^-1^ | Metal ions/  mol L^-1^ | Formula |
| --- | --- | --- | --- | --- |
| VCo-PBA | 0.24774 | 0.16379 | 0.03781 | V_0.753_Co_0.115_Fe(CN)_6_·4.44H_2_O |
| VCu-PBA | 0.24146 | 0.16807 | 0.03231 | V_0.741_Cu_0.100_Fe(CN)_6_·4.74H_2_O |
| VMn-PBA | 0.2583 | 0.19548 | 0.05673 | V_0.604_Mn_0.458_Fe(CN)_6_·4.61H_2_O |
| VNi-PBA | 0.23852 | 0.18279 | 0.06765 | V_0.624_Ni_0.480_Fe(CN)_6_·4.51H_2_O |
| VFe-PBA | 0.21614 | 0.31393 | 0 | V_0.741_Fe_1.08_(CN)_6_·4.52H_2_O |
| VZn-PBA | 0.11743 | 0.3675 | 0.39992 | V_0.300_Zn_1.02_Fe(CN)_6_·5.98H_2_O |

**Figure S14**. High-resolution XPS spectra of VCo-PBA. (a) V 2p. (b) Fe 2p. (c) C 1s. (d) N 1s. (e) O 1s.

**Figure S15**. High-resolution XPS spectra of VCu-PBA. (a) V 2p. (b) Fe 2p. (c) C 1s. (d) N 1s. (e) O 1s.

**Figure S16**. High-resolution XPS spectra of VMn-PBA. (a) V 2p. (b) Fe 2p. (c) C 1s. (d) N 1s. (e) O 1s.

**Figure S17**. High-resolution XPS spectra of VNi-PBA. (a) V 2p. (b) Fe 2p. (c) C 1s. (d) N 1s. (e) O 1s.

**Figure S18**. High-resolution XPS spectra of VFe-PBA. (a) V 2p. (b) C 1s. (c) N 1s. (d) O 1s.

**Figure S19**. High-resolution XPS spectra of VZn-PBA. (a) V 2p. (b) Fe 2p. (c) C 1s. (d) N 1s. (e) O 1s.

**Figure S20**. The GCD curves of VM-PBAs cathodes at the current density of 0.2 A g^-1^. (a) VCo-PBA. (b) VCu-PBA. (c) VMn-PBA. (d) VNi-PBA. (e) VFe-PBA. (f) VZn-PBA.

**Figure S21**.The dQ/dV curves of VCo-PBA at different cycles.

**Figure S22**.The dQ/dV curves of the 6^th^ cycle for VM-PBAs cathodes at the current density of 0.2 A g^-1^. (a) VCo-PBA. (b) VCu-PBA. (c) VMn-PBA. (d) VNi-PBA. (e) VFe-PBA. (f) VZn-PBA.

**Figure S23**. The cycling performance of VM-PBAs cathodes at the current density of 1 A g^-1^. (a) VCo-PBA. (b) VCu-PBA. (c) VMn-PBA. (d) VNi-PBA. (e) VFe-PBA. (f) VZn-PBA.

**Figure S24**. The GCD curves of VM-PBAs cathodes at the current density of 1 A g^-1^. (a) VCo-PBA. (b) VCu-PBA. (c) VMn-PBA. (d) VNi-PBA. (e) VFe-PBA. (f) VZn-PBA.

**Figure S25**. CV curves of the as-prepared VM-PBAs electrodes at the scan rate of 0.1 mV s^-1^. (a) VCo-PBA. (b) VCu-PBA. (c) VMn-PBA. (d) VNi-PBA. (e) VFe-PBA. (f) VZn-PBA.

**Figure S26**.The CV curves of M-PBAs cathodes at the scan rate of 0.1 mV s^-1^. (a) Co-PBA; (b) Cu-PBA; (c) Mn-PBA and (d) Ni-PBA;

**Figure S27**. The comparisons of the first cycle of CV curves between VM-PBAs and M-PBAs (M=Co, Cu, Mn and Ni) cathodes at the scan rate of 0.1 mV s^-1^

**Figure S28**. Log (𝑖) vs. log (𝑣) plots from the CV results at different scan rate.

**Figure S29**. In situ XRD results for VMn-PBA cathode from 5-80^o^. The marked peaks represent different crystal planes of PBA.

**Figure S30**. The viscosity data of the electrolytes.

**Figure S31**. ATR-FTIR spectra of glycerol-water electrolytes.

**Figure S32**. Soak an equal number of VM-PBAs in an equal amount of mixed liquid, and observe the UV spectrum of the supernatant.

**Figure S33**. The cycling performance of VM-PBAs cathodes at the current density of 1 A g^-1^ with the GE-5 electrolyte. (a) VCo-PBA. (b) VCu-PBA. (c) VMn-PBA. (d) VNi-PBA. (e) VFe-PBA. (f) VZn-PBA.

**Figure S34**. The GCD curves of VM-PBAs cathodes at the current density of 1 A g^-1^ with the GE-5 electrolyte. (a) VCo-PBA. (b) VCu-PBA. (c) VMn-PBA. (d) VNi-PBA. (e) VFe-PBA. (f) VZn-PBA.

**Figure S35**. The cycling performance of VM-PBAs cathodes at the current density of 1 A g^-1^ with the GE-4 electrolyte. (a) VCo-PBA. (b) VCu-PBA. (c) VMn-PBA. (d) VNi-PBA. (e) VFe-PBA. (f) VZn-PBA.

**Figure S36**. The cycling performance of VM-PBAs cathodes at the current density of 1 A g^-1^ with the GE-2 electrolyte. (a) VCo-PBA. (b) VCu-PBA. (c) VMn-PBA. (d) VNi-PBA. (e) VFe-PBA. (f) VZn-PBA.

**Figure S37**. The cycling performance of VM-PBAs cathodes at the current density of 0.5 A g^-1^ with the GE-2 electrolyte. (a) VCo-PBA. (b) VCu-PBA. (c) VMn-PBA. (d) VNi-PBA. (e) VFe-PBA. (f) VZn-PBA.

**Figure S38**. The GCD curves of VM-PBAs cathodes at the current density of 0.5 A g^-1^ with the GE-2 electrolyte. (a) VCo-PBA. (b) VCu-PBA. (c) VMn-PBA. (d) VNi-PBA. (e) VFe-PBA. (f) VZn-PBA.

**Figure S39**. The dQ/dV curves of VM-PBAs cathodes with the GE-2 electrolyte. (a) VCo-PBA. (b) VCu-PBA. (c) VMn-PBA. (d) VNi-PBA. (e) VFe-PBA. (f) VZn-PBA.

**Figure S40**. The cycling performance with VCu-PBA cathodes with different electrolytes at the current density of 1 A g^-1^.

**Figure S41**. The cycling performance with VNi-PBA cathodes with different electrolytes at the current density of 1 A g^-1^.

**Figure S42.** The cycling performance with VMn-PBA cathodes with GE-4 electrolyte at the current density of 2 A g^-1^.

**Figure S43.** The rate capability of VM-PBAs cathodes with the GE-4 electrolyte. (a) VCo-PBA. (b) VCu-PBA. (c) VMn-PBA. (d) VNi-PBA. (e) VFe-PBA. (f) VZn-PBA (Unit: A g^-1^).

**Figure S44.** The characterization of VMn-PBA after cycling when use the GE-4 electrolyte. (a) The SEM images of cathode after short term cycles. (b) The SEM images of cathode after long term cycles. (c) The comparison of XRD. (d) The comparison of XPS survey.

**Table S3.** The comparison of electrochemical performance of reported articles.

| Material | Electrolyte | Working window | Capacity (mAh g^-1^@A g^-1^) | Cycle life (cycles, retention%@A g^-1^) | Ref. |
| --- | --- | --- | --- | --- | --- |
| Zn_3_[Fe(CN)_6_]_2_ | 3 M ZnSO_4_ | 0.8-1.9 | 66.5@0.06 | 200, 81@0.3 | [1] |
| Zn_3_[Fe(CN)_6_]_2_ | 1 M ZnSO_4_ | 0.8-1.9 | 65.4@0.06 | 200, 80@0.3 | [2] |
| K_0.14_Mn_0.11_Zn_1.49_[Fe(CN)_6_] | 1 M ZnSO_4_ | 1.0-2.0 | 41.6@0.05 | 500, 85.5@0.25 | [3] |
| KCuFe(CN)_6_ | 1 M ZnSO_4_ | 0.2-1.2 | 56@0.05 | 20, 77@0.02 | [4] |
| NiFe(CN)_6_ | 0.5 M Na_2_SO_4_+0.05 M ZnSO_4_ | 0.9-1.9 | 76.2@0.1 | 1000, 81@0.5 | [5] |
| K_1.6_Mn_1.2_Fe(CN)_6_ | 2 M Zn(ClO_4_)_2_ in tetraethylene glycol dimethyl ether | 0.7-1.9 | 65@0.05 | 8500, 94@0.2 | [6] |
| K_2_MnFe(CN)_6_ | 30 M KFSI+1 M Zn(CF_3_SO_3_)_2_ | 0.5-1.9 | 100@0.2 | 400, 72@0.2 | [7] |
| CoMn-PBAHSs | 2 M Zn(CF_3_SO_3_)_2_ | 0.5-1.8 | 128.6@0.05 | 300, 76.4@10 | [8] |
| V_3_[Fe(CN)_6_]_2_ | 4 M Zn(CF_3_SO_3_)_2_ | 0.4-1.8 | 160@0.5 | 1000, 87.8@2 | [9] |
| ZnHCF/PANI | 7.5 M ZnCl_2_+4 M NaCl | 0.8–2.1 | 50@0.05 | 350, 75@0.5 | [10] |
| VMn-PBA | 0.4 g glycerol + 0.6 g H_2_O 3 m Zn(CF_3_SO_3_)_2_ | 0.2-2.0 | 110@1 | 1200, 80.2@2 | This work |

**Figure S45**. Lighting LED experiment

Reference

[1]	L. Zhang, L. Chen, X. Zhou, Z. Liu, Scientific Reports **2015**, 5, 18263.

[2]	L. Zhang, L. Chen, X. Zhou, Z. Liu, Advanced Energy Materials **2015**, 5, 1400930.

[3]	G. Ni, X. Xu, Z. Hao, W. Wang, C. Li, Y. Yang, C. Zhou, L. Qin, W. Chen, X. Yao, J. Cai, ACS Applied Energy Materials **2021**, 4, 602-610.

[4]	Z. Jia, B. Wang, Y. Wang, Materials Chemistry and Physics **2015**, 149-150, 601-606.

[5]	K. Lu, B. Song, J. Zhang, H. Ma, Journal of Power Sources **2016**, 321, 257-263.

[6]	Q. Li, K. Ma, G. Yang, C. Wang, Energy Storage Materials **2020**, 29, 246-253.

[7]	W. Deng, Z. Li, Y. Ye, Z. Zhou, Y. Li, M. Zhang, X. Yuan, J. Hu, W. Zhao, Z. Huang, C. Li, H. Chen, J. Zheng, R. Li, Advanced Energy Materials **2021**, 11, 2003639.

[8]	Y. Zeng, X. F. Lu, S. L. Zhang, D. Luan, S. Li, X. W. Lou, Angewandte Chemie International Edition **2021**, 60, 22189-22194.

[9]	Y. Zhang, Y. Wang, L. Lu, C. Sun, D. Y. W. Yu, Journal of Power Sources **2021**, 484, 229263.

[10]	Q. Liu, Z. Ma, Z. Chen, M. Cui, H. Lei, J. Wang, J. Fei, N. He, Y. Liu, Q. Liu, W. Li, Y. Huang, Chemical Communications **2022**, 58, 8226-8229.
